# Unveiling the Interactions of Doxorubicin with the Lipid Components of Liposomes for Its Delivery

**DOI:** 10.1021/acs.jpcb.5c00523

**Published:** 2025-05-06

**Authors:** Julia Alvarez-Malmagro, Lorena Ruano, María Cuartero-González, Juan J. Nogueira, Francisco Prieto-Dapena

**Affiliations:** † Departamento de Química Física, Facultad de Química, 16778 Universidad de Sevilla, 41012 Sevilla, Spain; ‡ Departamento de Química, Facultad de Ciencias, 16722 Universidad Autónoma de Madrid, 28049 Madrid, Spain; § Institute for Advanced Research in Chemistry (IAdChem), Universidad Autónoma de Madrid, 28049 Madrid, Spain

## Abstract

The characterization of drug/lipid interactions is key to developing novel and more efficient drug delivery systems. In this work, we combine electrochemical measurements, attenuated total reflection (ATR) spectroscopy, and molecular dynamics simulations to unveil the interacting mechanisms of doxorubicin (DOX) with lipid monolayers and bilayers containing a cytidine derivative nucleolipid, which serve as a model system of previously developed liposomes for DOX delivery. The nucleolipid was included in the liposome formulation to take advantage of its molecular recognition capabilities and its capacity to anchor gold nanoparticles. The compression isotherms of the Langmuir monolayers and interfacial capacitance measurements on a gold electrode modified with hybrid bilayers in the presence of DOX demonstrate the interaction of the drug with the nucleolipid polar heads. This is confirmed by computational simulations of a solvated DOX/bilayer complex, which show that the adsorption process is driven by stacking and electrostatic interactions involving the aromatic and nonaromatic moieties of DOX, respectively. Moreover, both ATR spectra of supported bilayers on silicon and simulations show that the presence of DOX does not significantly affect the tilt angles of the lipids. The system studied in this work is a promising therapeutic option for cancer treatment. The combined methodology applied to this study can serve as a reference for other studies of drug–carrier interactions.

## Graphical abstract

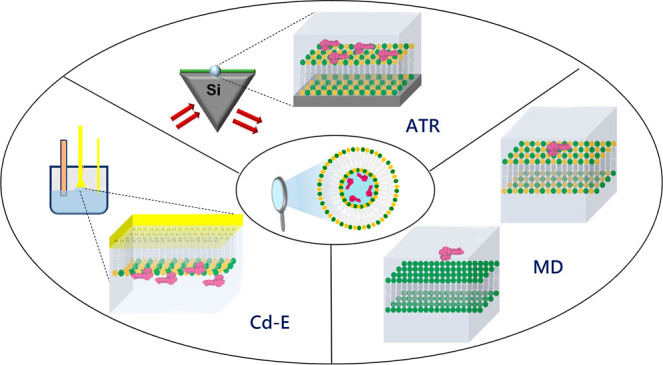

## Introduction

Doxorubicin (DOX), Scheme 1, is a first-generation anthracycline drug with a broad spectrum of activity against solid tumors and hematological neoplasms, constituting a key element in several clinical protocols for cancer treatment.^1–4^

**Scheme 1:**
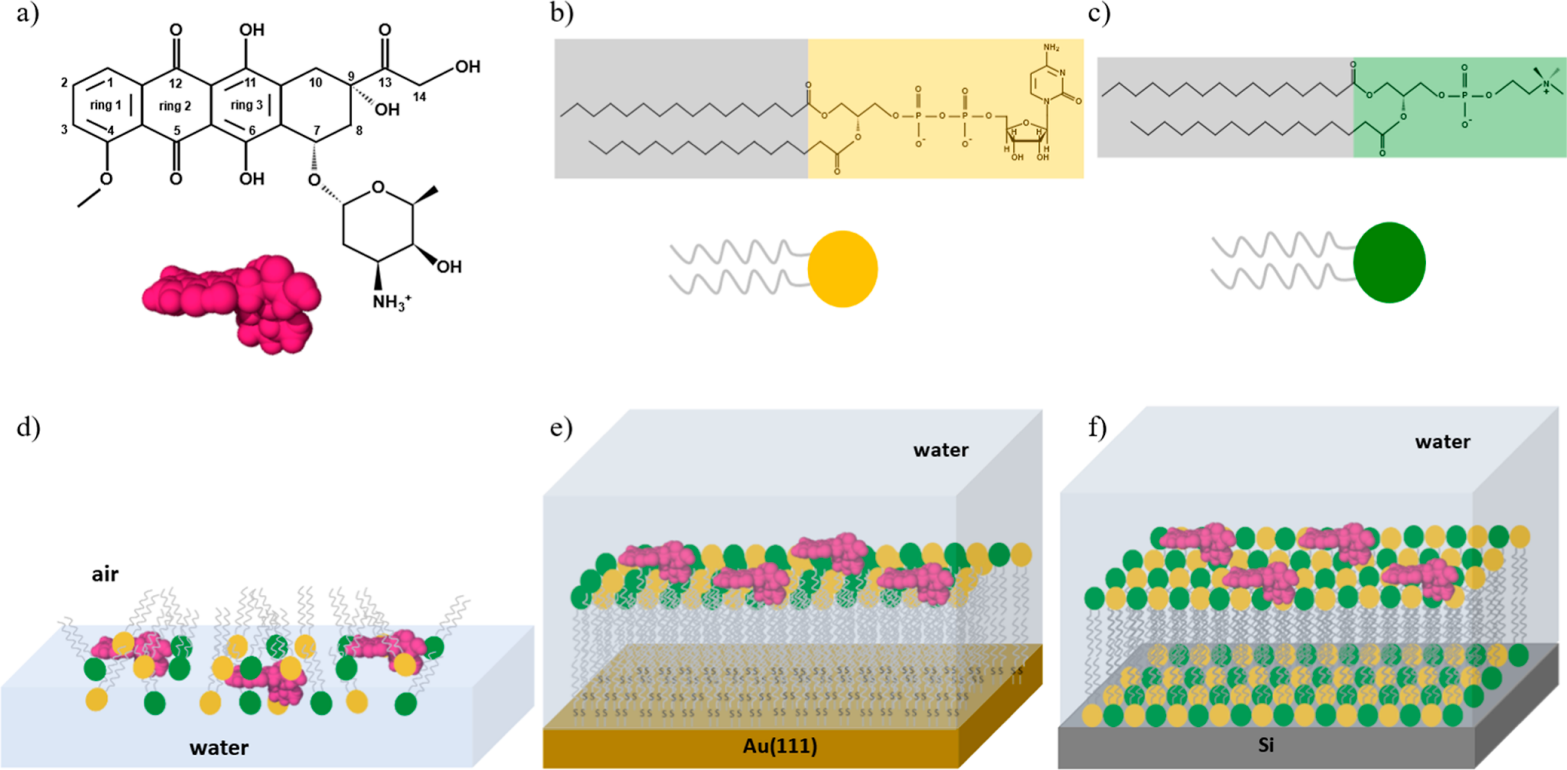
Molecular Structure of (a) Doxorubicin, (b) 1,2-Dipalmitoyl-*sn*-glycero-3-cytidine Diphosphate (DG-CDP), and (c) 1,2-Dipalmitoyl-*sn*-glycero-3-phosphocholine (DPPC); (d) Monolayers at the Air/Water Interphase; (e) Hybrid Bilayers on Au(111) and (f) Supported Bilayers on Si of Mixed DPPC:DG-CDP Lipid Films in the Presence of DOX

DOX cytotoxic activity occurs through a combined mechanism that includes intercalation between the nucleobases of double-stranded deoxyribonucleic acid (DNA), particularly the cytosine–guanine pairs, and the blockage of topoisomerase-II, thus inhibiting nuclear DNA replication.^5,6^ Many efforts have been made to improve the clinical utility of this drug, whose administration conventional therapy may prevent proper dosing and rechallenge in the case of relapse or lead to drug resistance. High cumulative doses of DOX increase the likelihood of cardiotoxicity, while individual doses are often limited by myelosuppression. The technology applied to the commercialized liposomal DOX formulations (Doxil, Caelyx, Myocet, and Lipo-Dox) combines pegylated liposomes to stabilize them sterically with the aim of increasing the half-life in circulation, and the DOX loading methodology by remote transmembrane loading, allowing optimization of the administration dose.^7^

Currently, research is focused on developing better drug delivery platforms. One of the most used alternatives in the specific case of DOX consists of the use of liposomal platforms with anchored gold nanoparticles (AuNPs) for the encapsulation and release of the drug, improving the therapeutic effects and reducing side effects due to the characteristics that they present. Liposomes favor the encapsulation of the drug in the aqueous nucleus and its release in the tumor cells,^8^ and the AuNPs are capable of generating a surface plasmon that increases the temperature of the environment when receiving IR radiation of appropriate frequency, increasing the fluidity of the lipid film and facilitating the localized release of DOX contained in the liposomal platform.^9^ In recent years, continuous advances in gene therapy have led to the use of structures such as short-chain oligonucleotides as therapeutic molecules of great application in pathologies, e.g., cancer, since they can interfere with pathological cells by silencing specific genes.^10^ The potential of this approach is enormous, but targeting and cellular internalization after administration represent major obstacle.

An alternative is the use of liposomes that incorporate nucleolipids,^11,12^ or lipid–oligonucleotides, in order to get advantage of their molecular recognition of complementary DNA or RNA strands and to improve cellular uptake.^13,14^ In a previous work, liposomes for DOX delivery were prepared, containing the nucleolipid 1,2-dipalmitoyl-*sn*-glycero-3-cytidine diphosphate (DG-CDP) and 1,2-dipalmitoyl-*sn*-glycero-3-phosphocholine (DPPC) in the lipid bilayer (see Scheme 1a–f) and anchoring PEGylated gold nanoparticles on the outer surface of liposomes, in order to take advantage of their photothermal properties for the controlled release of the DOX cargo.^15^ The mole fractions of the lipidic components were optimized by means of thermodynamic analysis of monolayers at the air/solution interphase in the Langmuir trough, and the structural characteristics of the lipid film and its molecular recognition capabilities toward the DNA complementary base, guanine, were studied by photon-modulated infrared reflection–absorption spectroscopy (PM-IRRAS).^16,17^ Kinetics release studies of DOX from the prepared liposomes revealed a fast controlled release, triggered by the temperature and/or light. The recovery of the DOX cargo was not complete, suggesting that the drug is partially retained by any of the liposome components. Cytotoxic tests of this formulation over cell cultures indicated that the interaction of the drug with liposome components does not affect the activity of the drug released,^15^ although it is not clear if it affects the activity of the retained fraction or what are the components of the lipid membrane involved in the interaction with DOX.

The availability of sensitive techniques to obtain structural information related to the interactions between liposomes and solutes in their aqueous core is limited. On the contrary, planar lipid membranes constitute a biomimetic physic model of liposome membranes that permit the application of electrochemical, spectroscopic, and microscopic techniques to get structural information.^18–23^ In addition, planar membranes are commonly employed in computer simulations, especially bilayers, since the computational models are simpler and of smaller size than liposomic models.^24–27^ Structural and energetic computational analyses enable rationalization of the experimental findings and prediction of the behavior of novel unexplored systems. Lipid monolayers at the air/water interphase of the Langmuir trough constitute a first approximation.^28^ The adsorption of species dissolved in the aqueous subphase can be followed by changes in the surface pressure.^29,30^ On the other hand, the compression elastic modulus obtained from the Langmuir isotherms can provide information about the influence that the presence of DOX can have in the fluidity of the lipid film, as it was pointed by Ceballos et al. in the study of toxicity mechanism of DOX using lipid formulations containing dipalmitoylphosphatidylserine (DPPS) and/or sphingomyelin,^31^ Matyszewska and Moczulska also studied the interaction of DOX with DPPS, including the effect of the pH, by the analysis of the Langmuir isotherms in combination to Brewster angle microscopy.^32^

Transfer of Langmuir monolayers to gold electrodes and the study of the electroreduction of DOX on these modified electrode surfaces have also been used to study the adsorption of DOX on the lipid membranes.^32,33^ However, the high number of defects present in these supported monolayers and the strong adsorption of DOX over gold surfaces made it difficult to separate DOX–gold interactions from DOX–lipid film interaction effects. An alternative that generates a lower number of defects is the use of lipid bilayers to modify electrode surfaces.^34,35^ These architectures are more realistic systems mimicking the lipid membranes in liposomes or living cells than supported monolayers. Different assemblies have been described in the literature, with distinct characteristics: supported bilayer lipid membranes, hybrid bilayer lipid membranes, tethered bilayer lipid membranes, and floating bilayer lipid membranes.^34,35^

Modification of gold electrode surfaces with model planar lipid bilayers allows the application of electrochemical techniques that provide information on the dielectric properties of the modified electrode, which are related to the ionic permeability and defects of the bilayer. Pseudocapacitance plots are useful to obtain information on the homogeneity of the lipid film modifying the electrode. In addition, internal reflection FT-IR spectroscopy in the attenuated total reflection (ATR) mode of artificial biomimetic membranes provides information about the structure of lipid films over solid substrates and the interactions with incorporated biomolecules.^36,37^

The aim of this work is to study the possible interactions between DOX and the components of the lipid vesicles used in a previous work for its delivery, including the cytidine derivative nucleolipid. Measurements of the Langmuir isotherms of lipid monolayers at the air/aqueous buffer interface provide initial evidence about the interaction of DOX with the components of the liposome wall. The modification of Au(111) electrodes with lipid bilayers allows us to measure the pseudocapacitance of the lipid film-modified electrode, in the absence and presence of DOX, and to determine if the DOX interaction with the lipid film induces the formation of defects. ATR measurements of lipid bilayers supported on the Si surface provide information about the tilt and twist angles of the lipid acyl chains, which can be related to the influence of DOX in the stability of the vesicles’ walls. Finally, classical molecular dynamics simulations have been performed to complement the experiments and reveal the nature of the intermolecular interactions that govern the adsorption process of DOX.

## Materials and Methods

### Reagents and Solutions

All reagents were purchased in suprapure grade from Sigma-Aldrich, Merck, or Avanti Polar Lipids and used as received. All aqueous solutions were prepared by employing ultrapure water purified with a Milli-Q system (Merk-Millipore).

Stock solutions 1 mg·mL^–1^ 1,2-dipalmitoyl-*sn*-glycero-3-phosphocholine (DPPC, Avanti Polar Lipids) and 1 mg·mL^–1^ 1,2-dipalmitoyl-*sn*-glycero-3 cytidine diphosphate (DG-CDP, Avanti Polar Lipids) were prepared in chloroform (Sigma-Aldrich, analytical grade). Stock solutions of the 3:7 mixture in the molar fraction of DPPC:DG-CDP were prepared by mixing the required volumes of the previous stock solutions. Lipid solutions and their mixtures were stored at −20 ± 1 °C. The stock solution of 1-hexadecanethiol (Merck) was prepared in methanol and stored at 4 ± 1 °C.

Solutions based on 0.1 M NaF (Sigma-Aldrich—BioXtra, 99%) at pH 8 and stock solutions of 2.41 mM doxorubicin hydrochloride (DOX, Sigma-Aldrich) were prepared in deuterium oxide (Sigma-Aldrich 99.99%) for infrared measurements and in ultrapure water for Langmuir–Blodgett experiments. Stock solutions of DOX were kept at 4 ± 1 °C under light protection and employed only for 2 weeks to ensure their good stability.

General glassware was cleaned by overnight immersion in an acid solution of K_2_MnO_4_, washed afterward with dilute piranha solution, and thoughtfully rinsed with ultrapure water. Vials employed to prepare lipid stock solutions were cleaned by overnight immersion in a concentrated piranha solution (3:1 H_2_SO_4_/H_2_O_2_, v/v) and thoughtfully rinsed with ultrapure water.

### Langmuir Trough Measurements

Surface pressure versus area per molecule isotherm (π – *A*) as well as the surface pressure time curves (π vs *t*) were recorded on a Nima 611D Langmuir trough equipped with two mobile hydrophilic barriers of PTFE, a PS4 pressure sensor, and a filter paper Wilhelmy plate. The total volume of the trough is 150 mL, and the surface is 270 cm^2^. The trough is placed inside a Perspex urn to avoid contamination and interference. All measurements were carried out at 25 ± 1 °C employing either water or 0.1 M NaF as a subphase.

Once the mobile barriers and the Langmuir trough were cleaned with successive washing with methanol and Milli-Q water, a lipid monolayer in the air/aqueous solution interface was prepared. For that, 150 mL of aqueous phase, containing or not 10 μM DOX, is added to the Langmuir trough. Then, with the barriers open, a volume between 15 and 30 μL of the desired lipid solution is added on the subphase, and the chloroform is allowed to evaporate for 20 min. Finally, the barriers were compressed at a speed of 20 cm^2^·min^–1^, while the surface pressure (π) is recorded. All isotherms were repeated at least 2 times to ensure reproducibility.

In surface pressure–time (π vs *t*) measurements, the lipid monolayer formed at the air/aqueous solution interface was compressed to the desired initial surface pressure. Then, a volume of DOX stock solution was injected in the subphase to achieve the desired concentration, and changes in the surface pressure with time with a constant area of the monolayer were recorded.

### Preparation of Mixed Vesicles DPPC:DG-CDP(3:7)

The appropriate amount of stock solutions of DPPC and DG-CDP in chloroform was added to a glass test tube to obtain a lipid mixture of DPPC:DG-CDP 3:7 ratio in mole fractions. The resulting solution was gently shaken with a vortex under an argon atmosphere to slowly evaporate the chloroform. As a result, a dry film of the lipid mixture was formed and kept in a desiccator for at least 24 h before being used.

Vesicle formation was carried out at 50 ± 1 °C, a temperature higher than the phase transition temperature of DPPC (42 °C). An appropriate volume of 0.1 M NaF solution was added to the glass test tube to achieve a final concentration of 1 mg·mL^–1^ lipid mixture. The mixture was then sonicated for 30 min at 50 ± 1 °C to ensure the formation of vesicles with an appropriate size homogeneously distributed in the solution.

### Capacitance Measurements of Hybrid Bilayers on Au(111)

The capacitance–potential curves were obtained from the component imaginary of the electrochemical impedance measured at 25 Hz with an AC amplitude of 10 mV, with a PGSTAT 30 device from AutoLab.

For capacitance measurements, a Au(111) electrode was modified with a hybrid bilayer. First, the Au(111) electrode was cleaned by flame annealing several times and cooling for 3-4 minutes in an argon atmosphere. After that, a first self-assemble monolayer of 1-hexadecanethiol was formed by immersion of the Au(111) electrode in a 1 mg·mL^–1^ solution of the hexadecanethiol in ethanol for at least 12 h at 50 ± 1 °C to ensure complete coverage of the surface. Then, modified Au(111) was rinsed with ethanol and with Milli-Q water. Finally, to couple the second monolayer consisting of a DPPC:DG-CDP lipid mixture (3:7), a Langmuir–Schaefer (LS) transfer was carried out in the Langmuir trough.^34^

The impedance measurements have been carried out with a modified Au(111) electrode, as a working electrode (WE), in the presence and absence of 10 μM DOX in a 0.1 M NaF aqueous solution. A saturated electrode mercury/mercurous sulfate connected to the cell by a salt bridge that was filled with the same supported electrolyte solution was employed as a reference electrode. A gold wire flame-annealed was employed as a counter electrode. All potential values in this paper have been referenced versus the saturated calomel electrode (SCE).

50 mL portion of NaF aqueous solution, employed as a supporting electrolyte, was added into the electrochemical cell and purged with argon for 20 min. After that, an Ar blanket was left over the closed system to ensure an inert atmosphere. The contact between the electrolyte and the WE was made by the meniscus method^38^ to ensure that only the face with crystallographic orientation (111) had contact with the solution.

### Fourier Transform Infrared Spectroscopy

ATR measurements of supporting bilayers of DPPC:DG-CDP(3:7) on Si prism have been recorded with a NICOLET 6700 spectrophotometer equipped with an MCT-A detector, cooled with liquid N_2_, and with a VeeMax-II accessory from PIKE Technologies for the reflectance measurements. The infrared cell with a silicon prism placed at 60° as a reflection IR window was employed. The spectra were collected with lights s or p polarized selected using a ZnSe polarizer. Once the infrared cell is assembled, 200 μL of 0.1 M NaF electrolyte in D_2_O solution was added to the cell, and the reflectance spectra of the electrolyte with p and s polarized light were recorded. Supported bilayers were formed on the surface of the silicon prism. The prism face was heated to 60 ± 1 °C. Then, mixed DPPC:DG-CDP(3:7) vesicles suspended in D_2_O were added and kept there for 2 h while the supported bilayer was formed. Finally, the solution was removed and rinsed repeatedly with a 0.1 M NaF solution in D_2_O to remove the excess of vesicles that had not fused. After that, reflectance spectra of the supported bilayer were recorded. Finally, the exact amount of stock solution of DOX was added to the cell in order to achieve the concentration of 100 μM. The reflectance spectra in the presence of DOX were recorded. For each spectrum, 100 interferograms with a resolution of 4 cm^–1^ were collected.

Absorption infrared spectra in solution were obtained in two ways. The IR transmission spectrum of vesicles resuspended from the lipid mixture of DPPC:DG-CDP(3:7) in D_2_O was recorded by using a cell equipped with two circular CaF_2_ windows separated by a 50 μM Teflon spacer. For 10 mM DOX in D_2_O, the cell, described above for ATR-SEIRAS measurements, was used but using a ZnSe prism at 45° as an optical window. In both cases, a total of 1000 interferograms were collected with a resolution of 4 cm^–1^.

### Computational Details

The two lipid bilayers were built with the help of the PACKMOL^39^ software. The first one is the pure DPPC membrane formed by 100 molecules of DPPC per layer, and the second is DPPC:DG-CDP(1:1) composed of 50 DPPC and 50 DG-CDP molecules per layer. The experimental ratio between both lipid types, DPPC:DG-CDP(3:7), was not employed because the system was found to be unstable during the simulation. Thus, the ratio closest to the experimental one showing stability was employed. Both membranes have a 25 Å of water thickness on each side and a concentration of 0.1 M NaCl in addition to the ions needed to neutralize the system. The simulation was performed with NaCl instead of NaF, as in the experiments, because the force field for NaCl is more reliable, and the electrostatic interactions are the same since the atomic charges are the same for both species. Then, a single DOX molecule was manually placed in the bulk solvent at 38 Å from the center of mass of the membrane. This leads to four systems, two with the solvated membranes without DOX and the other two with the DOX molecule loaded in the solvent. The DOX molecule was initially optimized with density functional theory (DFT) at the B3LYP/6-31-G* level of theory using Gaussian09 ^40^ software before loading it in the solvated membrane.

The DPPC lipid molecules were obtained from the CHARMM-GUI^41^ Web site, and they were described by the Lipid21 ^42^ force field. In the case of DG-CDP, the nucleotide was created by the NAB^43,44^ module of AmberTools22 ^45^ and was described by the OL3 ^46^ force field, and the rest of the molecule was taken from the DPPC lipid. The missing phosphate parameters were taken from ref 47. Water molecules were described by the TIP3P^48^ model, Na^+^ and Cl^–^ ions by the parameters developed by Joung and Cheatham,^49^ and the DOX molecule by the general Amber force field.^50^

The next step was to run molecular dynamics simulations to equilibrate the systems. The protocol was the same for the four systems, but those with DOX molecules include restrictions applied to these molecules. First, three consecutive minimizations were carried out using the steepest descent method for 5000 steps and the conjugated method for the following 5000 steps. Positional restraints with force constants of 25, 5, and 0 kcal/(mol Å^2^) were applied on the lipids in each of the minimizations and 25, 5, and 5 kcal/(mol Å^2^) on DOX. Then, the systems were heated to 300 K for 400 ps employing the Langevin thermostat with a collision frequency of 1 ps^–1^ in the *NVT* ensemble. Positional restraints were applied using a force constant of 10 kcal/(mol Å^2^) on lipids and 5 kcal/(mol Å^2^) on DOX. Once the systems were at the desired temperature, four consecutive simulations of 1 ns at constant pressure, *NPT* ensemble, were performed with positional restraints on the lipids by applying force constants of 10, 5, 1, and 0 kcal/(mol Å^2^), respectively, and 5 kcal/(mol Å^2^) on DOX for the four simulations. The Langevin thermostat with a collision frequency of 1 ps^–1^ and the Berendsen barostat with a pressure relaxation time of 1 ps were employed to maintain the systems at 300 K and 1 bar. Finally, a production of 500 ns in the *NPT* ensemble was performed without positional restrictions on lipids or DOX. For all the steps, the particle mesh Ewald method^51^ was used with a cutoff of 10 Å for computing the nonbonded interactions. Moreover, the hydrogen mass repartitioning^52^ approximation was employed allowing a time step of 4 fs, and bond distances involving hydrogen atoms were restrained by the SHAKE^53^ algorithm. All of the simulations described above were carried out by the AMBER22 ^45^ program. In order to check that the systems are equilibrated, the root-mean-square deviation (RMSD), which provides the difference of the geometry of the system at each step along the simulation with respect to the initial geometry, and the area per lipid were monitored and are represented in Figures S1 and S2, respectively. As can be seen, both properties are converged after 250 ns of simulation for the solvated bilayers without DOX and after 100 ns in the case of the membranes in the presence of the DOX molecule. The subsequent analyses have been performed for the part of the simulations where the systems are equilibrated.

The quantum mechanical calculation of the vibrational spectrum of DOX was performed using DFT with the B3LYP functional and the 6-311++G­(d,p) basis set, as implemented in Gaussian09,^40^ considering the solvent with the polarizable continuum model.

## Results and Discussion

### Compression Isotherms and Insertion Measurements on Langmuir Monolayers at the Air/Water Interphase

Figure 1 shows that the *A*_molec_ values of DG-CDP monolayers are higher than those of DPPC, at a given surface pressure, π. This indicates repulsion interactions between the polar heads and/or steric effects of the nucleolipid molecules, as the acyl chains of DPPC and DG-CDP are identical. The mixture DPPC:DG-CDP(3:7) exhibits similar values of *A*_molec_ at given surface pressures higher than 20 mN m^–1^ than the film of pure DPPC, while at low constant surface pressures, the area per lipid of DPPC:DG-CDP is slightly larger, in good agreement with the simulations (see Figure S2). In the presence of DOX in the subphase, the compression isotherms of the monolayers containing DG-CDP shift to a higher molecular area. On the contrary, the compression isotherm obtained for monolayers of pure DPPC does not change in the presence of DOX. The presence of DOX in the subphase also decreases the slopes of the compression isotherms of films containing nucleolipids in Figure 1, particularly at low surface pressure, indicating that the monolayers affected become more fluid in the presence of DOX. This can be better quantified by the elastic compression modulus, *C*_s_^–1^, defined in eq 1.

1 $$C_{s}^{-1}=-\frac{1}{A_{molec}}{\left(\frac{\partial A_{molec}}{\partial \pi }\right)}_{T}$$

**Figure 1:**
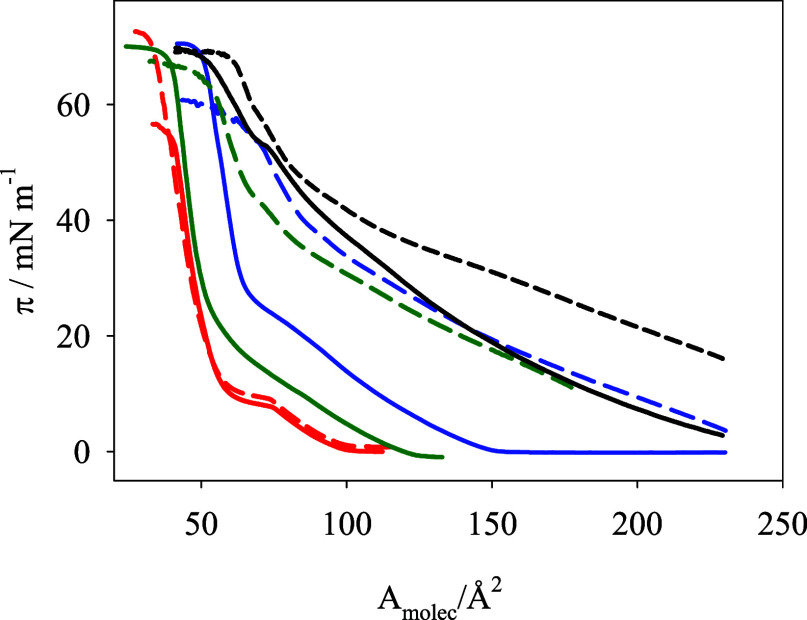
Compression isotherms, surface pressure (π) vs molecular area (*A*_molec_), measured at the air/water interphase at 24 °C with monolayers of pure DPPC (red lines), pure DG-CDP (blue lines), and mixtures DPPC:DG-CDP (3:7, optimum mole composition) (green lines) in the presence (dashed lines) and absence (solid lines) of DOX 10 μM in the subphase. Black lines represent the isotherms measured at the 0.1 M NaF/H_2_O interphase with a monolayer of DPPC:DG-CDP(3:7) in the absence (solid line) and presence (dashed line) of DOX 10 μM in the subphase.

Figure S3 shows the *C*_s_^–1^ vs π plots corresponding to the isotherms in Figure 1.^54^ The transitions between liquid-expanded and liquid crystalline states characterized by a minimum in *C*_s_^–1^ at ca. 20 mN m^–1^ shift to higher surface pressure and become less sharp in the presence of DOX, showing once again that the fluidity of the membrane increases. These results indicate that DOX interacts with the polar heads of the nucleolipid, as the acyl chains are identical for DPPC and DG-CDP molecules, and the presence of DOX only affects the films containing the nucleolipid. This agrees with the results found in the simulations in which the DOX molecule barely interacts with the lipid tails, but it strongly interacts with the heads of the nucleolipid, as explained below (see Figures 2 and 3).^17^

**Figure 2:**
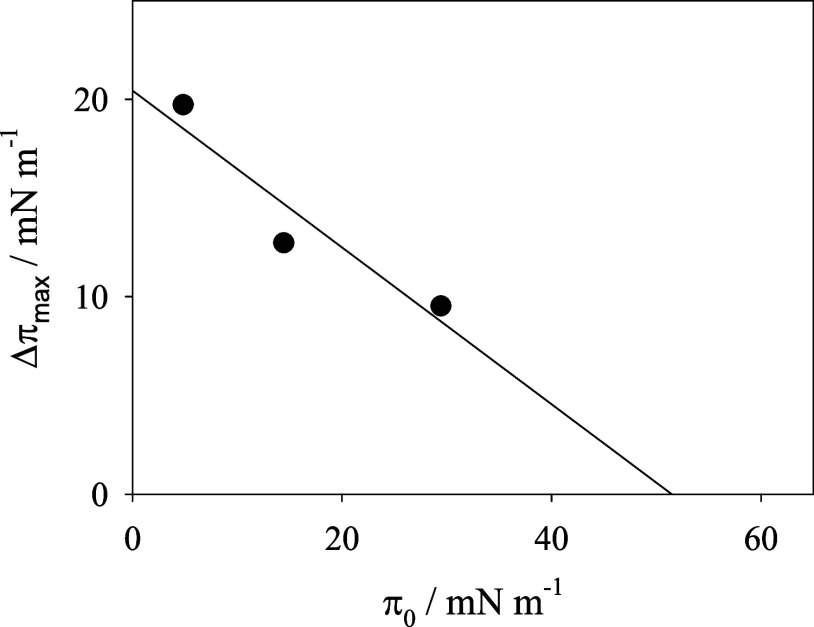
Δπ_max_ vs π_0_ plots corresponding to the insertion experiments in Figure S4.

**Figure 3:**
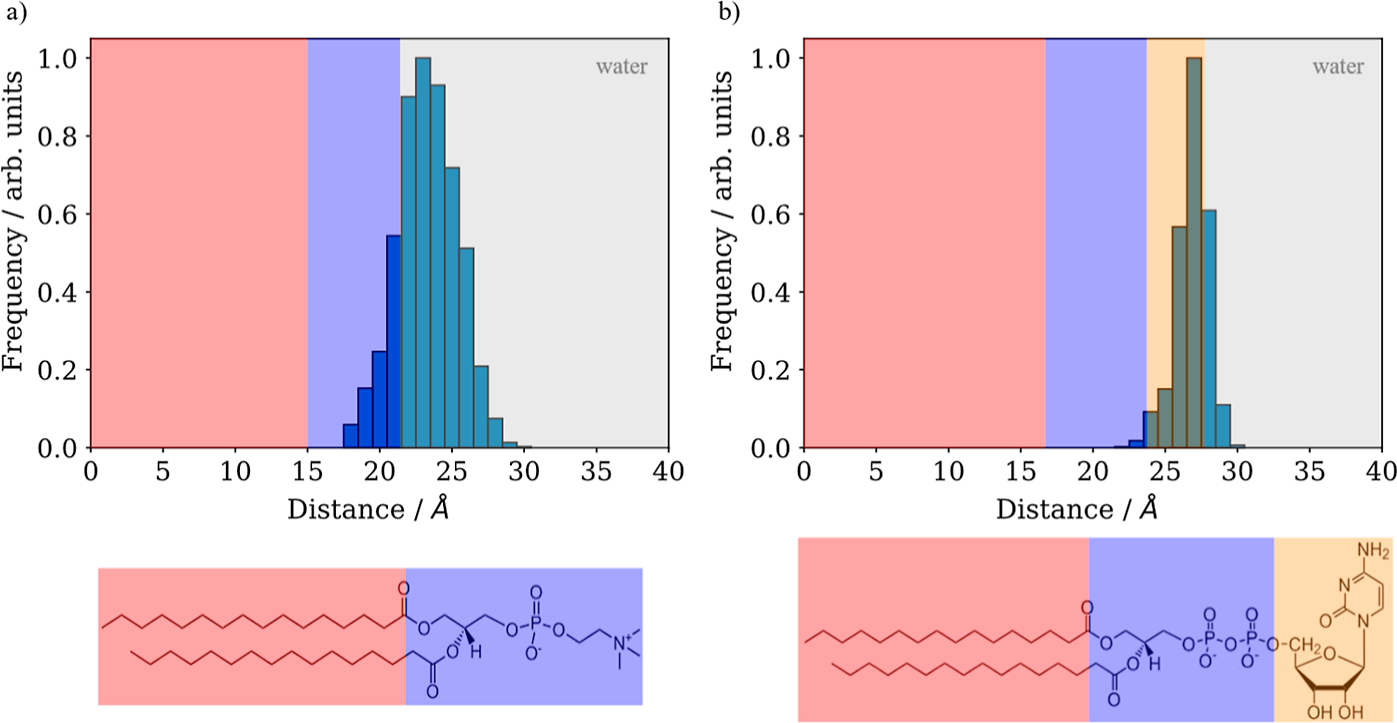
Probability distributions of distances between the center of mass of the nonaromatic ring of the DOX molecule and lipid bilayers along the converged part of the trajectory: (a) DPPC and (b) DPPC:DG-CDP(1:1). DPPC (down left) and DG-CDP (down right) lipids are represented with polar heads in blue, nonpolar tails in red, and nucleotide in orange. These colors are also in the plots, indicating the position of each fragment of the lipids.

With a subphase of 0.1 M NaF/H_2_O, the behavior is similar, although the area per molecule values are higher at a given surface pressure, as can be seen in Figure 1.

The inclusion of DOX in the monolayer containing a nucleolipid is confirmed by insertion kinetics experiments, measuring the increase of surface pressure of the monolayer at a constant molecular area as a function of time after the injection of DOX into the subphase, shown in Figure S4.

Experimental insertion plots can be explained with a pseudo-first-order kinetics model that provides the values of the maximum surface pressure increase (Δπ_max_) and the pseudo-first-order kinetic constant (β) as adjustable parameters.^55^ The plot of Δπ_max_ vs π_0_, in Figure 2, provides the exclusion surface pressure, from the abscissa intercept. The exclusion surface pressure is an estimation of the maximum surface pressure at which the adsorbate can interact with the film without affecting its stability. The value obtained, higher than 50 mN m^–1^, higher than the collapse pressure, confirms that DOX is adsorbed on the lipid film at any surface pressure, and it suggests that the adsorption does not alter the stability of the lipid monolayer.

The results obtained from the compression isotherms and inclusion of DOX with monolayers at the air/water (or aqueous solution) interphase constitute the evidence of the selective interaction of DOX with the nucleoside moieties of the nucleolipid in the monolayer. However, they do not provide information about the effect of DOX on the stability of liposome walls, formed by lipid bilayers with aqueous solutions at both sides.

### DOX/Bilayer Intermolecular Interactions by Molecular Dynamics Simulations

The adsorption of DOX on the lipid membranes was computationally observed by molecular dynamics simulations. The DOX molecule is spontaneously adsorbed along the simulations in both bilayers without the need to apply artificial forces that bias the dynamics. Figure 3 represents the distribution of distances between the center of mass of the nonaromatic ring of the DOX molecule and the 6 closest lipids that interact with DOX along the converged part of the trajectory. In other words, the distributions resemble the position of DOX along the *z*-axis normal to the bilayers, indicating the region of the membrane where the DOX is adsorbed. It can be noticed that in the case of the DPPC membrane (Figure 3a), the nonaromatic ring of the DOX molecule enters the polar heads’ region during part of the simulation time, but in most cases, it is closer to the water solvent. On the other hand, in the DPPC:DG-CDP(1:1) membrane (Figure 3b), the nonaromatic ring of DOX is most of the time in the region of the nucleotides of the lipid heads, indicating a stronger interaction of DOX with the DPPC:DG-CDP(1:1) membrane than with the DPPC one because of the presence of nucleotides.

Figure 4a,b shows the representative geometry of DOX interacting with the DPPC membrane and a representative geometry of a π-stacked orientation between DOX and the nucleolipids of the DPPC:DG-CDP(1:1) membrane, respectively. In the DPPC membrane (Figure 4a), only the nonaromatic ring of DOX is adsorbed; therefore, the interactions are mainly electrostatic, in part because of the formation of hydrogen bonds. These hydrogen bonds are mostly formed with the OH and NH_3_^+^ groups of the nonaromatic ring of the DOX molecule, as seen in Figure 4c, which contains the total fraction of time during which a specific group is involved in hydrogen bonding. Note that these time fractions can be higher than one if the same group is participating in more than one hydrogen bond per snapshot. For example, if an OH group is involved in a specific hydrogen bond during the whole simulation and in another hydrogen bond during half of the simulation, then the hydrogen bonding fraction of time for that OH group would be 1.5. In the case of the DPPC:DG-CDP(1:1) bilayer, not only the nonaromatic ring of DOX is adsorbed—it presents hydrogen bonds, although for less time than in the DPPC membrane (see Figure 4d)—but the aromatic part is adsorbed too because there are π-stacking interactions between the aromatic moiety of the DOX molecule and the nucleotides. In addition, the simulations show that the nucleotides that surround the DOX molecule are oriented upward favoring π-stacking interactions.

**Figure 4:**
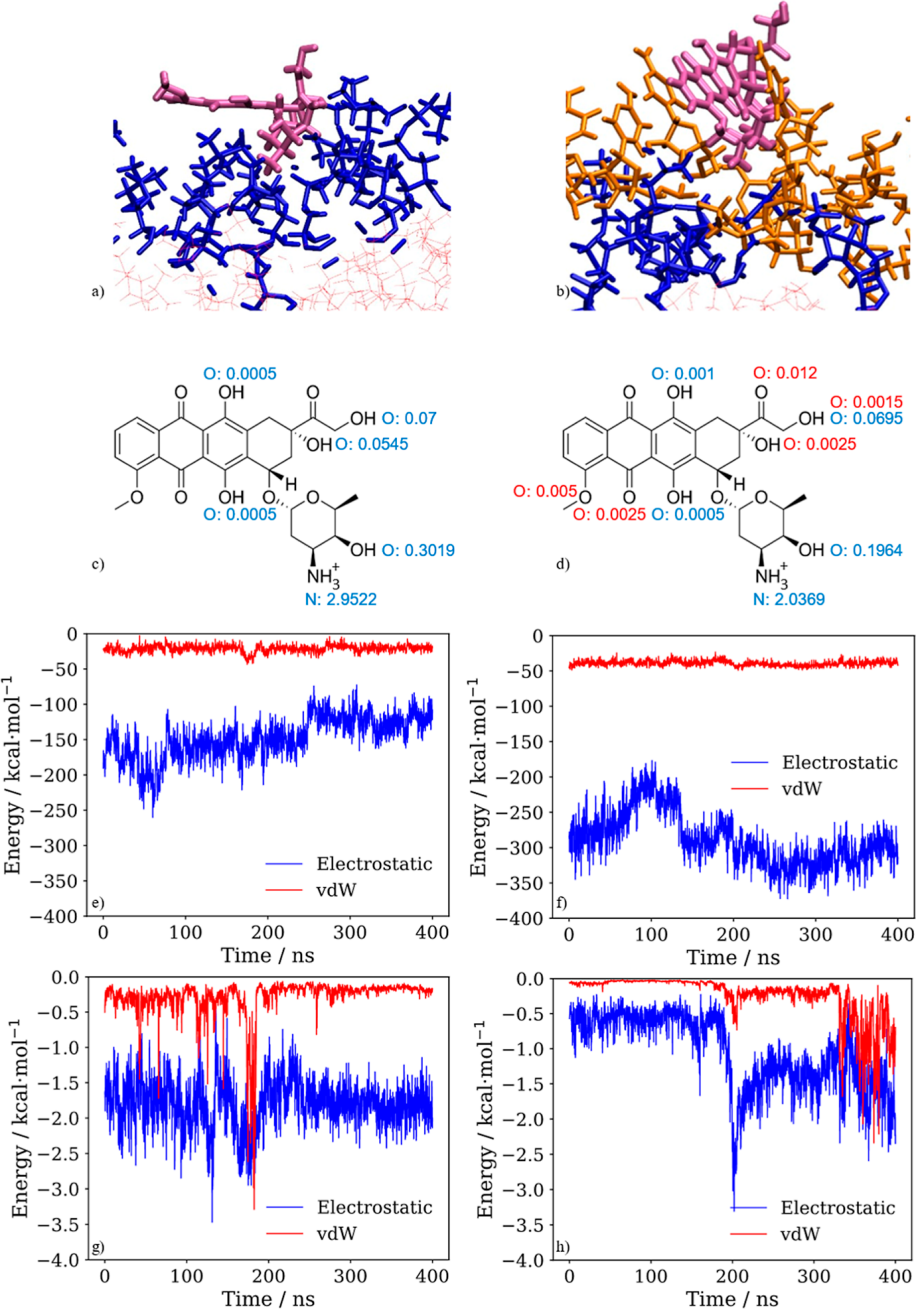
(a) Representative geometry of the DOX molecule in the DPPC membrane. (b) Geometry representing π-stacking interactions between the DOX molecule and DPPC:DG-CDP(1:1) membrane. Hydrogen bond analysis between the DOX molecule and DPPC membrane (c) and the DPPC:DG-CDP(1:1) bilayer (d). The numbers represent the sum of the fractions of time during which each hydrogen bond is present. Color code: H donor in blue and H acceptor in red. Time evolution of the interaction energy between the DOX molecule and the heads of (e) DPPC and (f) DPPC:DG-CDP(1:1) membranes, and between the DOX molecule and the tails of (g) DPPC and (h) DPPC:DG-CDP(1:1) lipid bilayers. Color code: DOX in mauve, polar heads in blue, nonpolar tails in red, and nucleotide in orange.

In order to further characterize these intermolecular interactions, the interaction energy between the DOX and the nucleolipid molecules within a sphere of 12 Å has been computed classically by the force field. Figure 4e,f displays the interactions between DOX and the polar heads of DPPC and DPPC:DG-CDP(1:1) lipids, respectively. In both cases, electrostatic interactions dominate, although in the DPPC:DG-CDP(1:1) membrane, they are stronger (higher in absolute value), meaning that the DOX molecule interacts in a stronger way with the DPPC:DG-CDP(1:1) membrane than with the DPPC bilayer. Moreover, the DOX/DPPC:DG-CDP(1:1) complex also has a stronger van der Waals contribution due to the π-stacking interactions. In the case of the interactions between the DOX molecule and the nonpolar tails (Figure 4g,h), there is not a great difference between the two membranes because DOX does not penetrate deep in the bilayers, although this contribution is slightly higher (in absolute value) in the DPPC membrane because the DOX molecule is closer to the nonpolar region than in the mixed bilayer.

### Pseudocapacitance Plots of Au(111) Electrodes Modified with Hybrid Lipid Bilayers

Figure 5 shows that the presence of the hybrid bilayer anchored on Au(111) diminishes the pseudocapacitance values obtained with the bare Au(111) in 0.1 M NaF. The *C*_ps_ minimum values (ca. 1.44 ± 0.01 μF cm^–2^) are close to the theoretical values corresponding to a perfect hybrid bilayer (0.8 μF cm^–2^). Moreover, there is not any peak at ca. 0.2 V vs SCE for the percolation of anions induced by the electric field. These findings confirm the formation of a hybrid bilayer on gold without defects. In the presence of DOX 10 μM, the minimum of capacitance slightly decreases to 1.35 ± 0.01 μF cm^–2^. This change could be explained if DOX interacts with the nucleolipid polar heads, slightly increasing the thickness of the dielectric layer covering the gold electrode. A calculation with a simple dielectric model considering that capacitance is proportional to the inverse of the thickness and that the dielectric constant does not change upon the addition of DOX provides a 7% averaged thickness increase with the adsorption of DOX, ca. around 3 Å.

**Figure 5:**
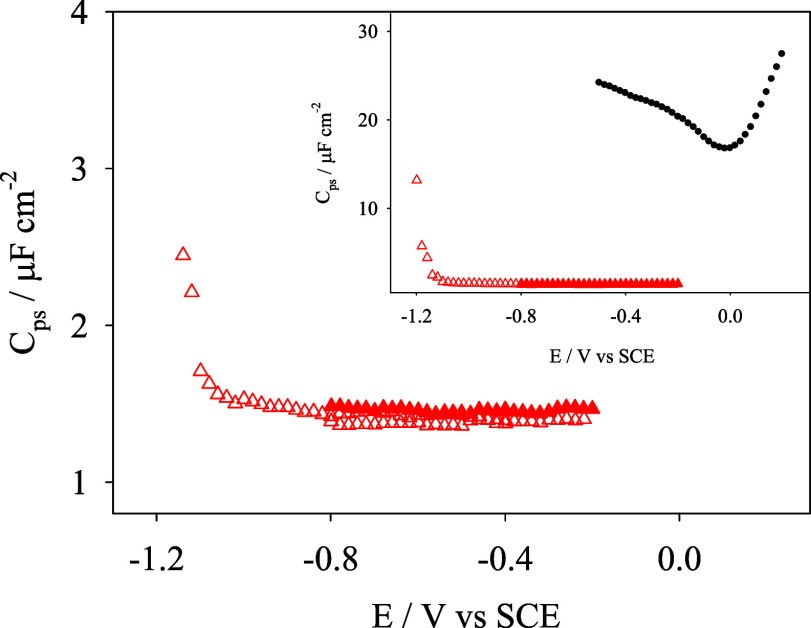
Pseudocapacitance (*C*_ps_) versus potential plots measured in NaF 0.1 M with Au(111) electrode-modified hybrid bilayers of 1-hexadecanethiol and DPPC:DG-CDP(3:7) (red triangles) in the absence (filled symbols) and presence (hollow symbols) of 10 μM DOX. The inset is the same graph with a larger scale to show with black circles the pseudocapacitance corresponding to the bare Au(111) electrode in NaF 0.1 M.

Moreover, the potential can be extended up to −1.2 V vs SCE without obtaining signals corresponding to DOX electroreduction (ca. −0.45 V vs SCE), indicating that at higher potentials, the bilayer can be considered impermeable to DOX and discarding any possible effect in the bilayer organization caused by the electroreduction of DOX. Only at potentials lower than −1.2 V vs SCE, at which the reductive desorption of 1-hexadecanethiol takes place, the capacitance values increase rapidly by the charge transfer associated with electroreduction of DOX.

### ATR of Bilayers of DPPC:DG-CDP(3:7) Supported on Si

The electrochemical results presented before suggest that the presence of DOX does not decrease the organization of the lipid film, induced by the hydrophobic interactions between acyl chains. To confirm this, ATR spectra of DPPC:DG-CDP(3:7)-supported bilayers formed by vesicle fusion over one face of a Si prism have been collected in the presence and absence of DOX in 0.1 M NaF, with radiation polarized in the reflection plane (light p) and in the normal plane (light s). The results, in the CH stretching spectral region, are shown in Figure 6a–c.

**Figure 6:**
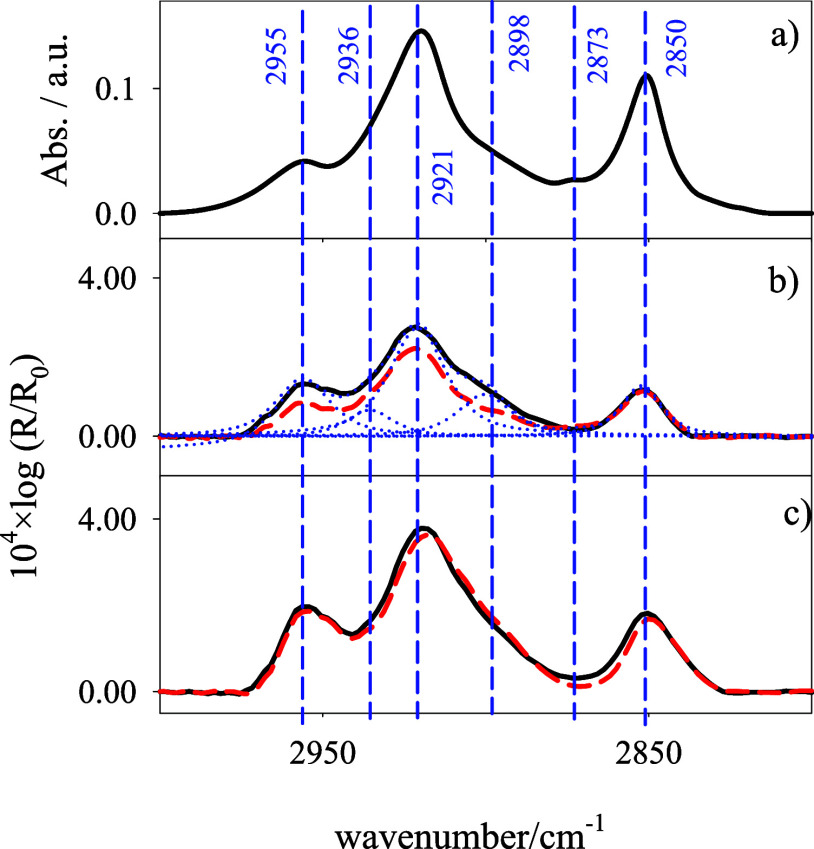
(a) Transmission FT-IR spectrum of DPPC:DG-CDP(3:7) liposomes in D_2_O. (b,c) ATR spectra in the CH stretching vibrational region of the DPPC:DG-CDP(3:7) bilayer supported on Si in contact with 0.1 M NaF/D_2_O in the absence (panel b) and presence of DOX 100 μM (panel c), measured with light p (black solid line) and light s (dashed red line). An example of the deconvolution of the spectra into individual vibrational bands is shown in (panel b). The blue vertical lines are the signal of different normal modes explained in the text.

The spectra have been deconvoluted into 6 individual spectral bands, whose assignment is indicated in Table S1. Bands at 2850 and 2921 cm^–1^ correspond to the symmetric and antisymmetric CH_2_ stretching of the lipid acyl chains, respectively. The position of these bands is typical of a lipid film in gel state with the acyl chains stretched in preponderant all-trans configuration, although with some contribution of gauche conformers.^56–58^

In ATR, the penetration depth of the evanescent wave, *d*_p_, can be expressed by^59^

2 $$d_{p}=\frac{\lambda }{2\pi \sqrt{n_{1}^{2}\sin^{2}\beta -n_{2}^{2}}}$$

with λ being the wavelength of the radiation, β being the incidence angle, and *n*_1_ and *n*_2_ being the refraction indexes of the incidence and reflection media, respectively. Then, according to eq 2, for ATR in the Si/water interphase with 60° of incidence angle, the penetration depth is several tenths of nanometers. For a lipid film located at the interphase, provided that its thickness is much lower than *d*_p_, the dichroic ratio *R*, the ratio between the absorption obtained with polarized light p and s, an order parameter of the transition dipole associated with the absorption can be calculated, *S*_dip_.^60–62^ Considering that the interphase is in plane *xy* and plane *xz* contains the incidence and reflected beams, as can be seen in Figure S6, *S*_dip_ can be expressed by

3 $$S_{dip}=\frac{E_{x}^{2}-RE_{y}^{2}+E_{z}^{2}}{E_{x}^{2}-RE_{y}^{2}-2E_{z}^{2}}$$

where *E*_*x*_, *E*_*y*_, and *E*_*z*_ are the electric field intensities in the *x*, *y*, and *z* directions, respectively.^60–62^ They can be calculated from the optical and geometrical properties of the system, as indicated in the Supporting Information. *S*_dip_ can provide the angle between the vibrational transition dipole moment and the normal direction to the interphase, θ_dip_:

4 $$\theta_{dip}=\cos^{-1}\sqrt{\frac{2S_{dip}+1}{3}}$$

Integrated intensities of the individual bands corresponding to ν_sim_(*CH*_2_) (2850 cm^–1^) and ν_asim_(*CH*_2_) (2921 cm^–1^) were used to calculate the dichroic ratio of both vibrations. With eqs 3 and 4, the values of the TDMs relative to the normal direction to the interphase, $\theta_{\nu_{sim}\left({CH}_{2}\right)}$ : and $\theta_{\nu_{asim}\left({CH}_{2}\right)}$ , have been calculated for the bilayer in the presence and absence of 100 μM in the supporting electrolyte. Table 1 contains these results.

**Table 1:** Averaged Angles in Degrees Relative to the Normal Direction of the Bilayer of the Transition Dipole Moments (TDMs) of ν_sim_(*CH*_2_) and ν_asim_(*CH*_2_) and Tilt Angle of the Acyl Chains of the Lipid Components in the Presence and Absence of DOX^a^

	$\theta_{\nu_{sim}\left(CH_{2}\right)}$	$\theta_{\nu_{asim}\left(CH_{2}\right)}$	θ_tilt_
bilayer	67 ± 3	62 ± 4	38 ± 6
bilayer + DOX	67 ± 3	67 ± 4	33 ± 5

a Errors in Table 1 have been estimated from two different runs, and they are mainly due to the baseline corrections.

ν_sim_(*CH*_2_) and ν_asim_(*CH*_2_) TDMs have directions perpendicular to each other and perpendicular to the axis of the acyl chain of the lipid molecule. Then, it is possible from geometrical considerations to deduce the expression in eq 5 that relates the tilt angle of the acyl chains relative to the normal to the interphase (θ_tilt_).^63–66^

5 $$\cos^{2}\theta_{tilt}+\cos^{2}\theta_{\nu_{sim}\left(CH_{2}\right)}+\cos^{2}\theta_{\nu_{asim}\left(CH_{2}\right)}=1$$

The results, included in Table 1, show that the tilt angle of acyl chains relative to the normal direction to the bilayer plane decreases in the presence of DOX, from 38 ± 6 to 33 ± 5°. Both values are in an excellent agreement to the value obtained for the average tilt angle of acyl chains in lipid monolayers of the same lipid mixture supported on Au(111) electrodes by PM-IRRAS, ranging from 34 to 40 depending on the electrode potential.^17^ The small change observed in the tilt angle in the presence of DOX indicates that it does not induce the disorder in the lipid film. On the contrary, it suggests a slightly better organization of the lipid molecules, making the averaged bilayer more compact in the presence of DOX, although the changes in the tilt angle of the acyl chains are within the precision of the calculation. This tilt angle range, 33–38°, is within the expected values for a liquid crystal phase of a lipid bilayer of DPPC.^60,67^

To computationally analyze the order of the lipid membranes, the deuterium order parameter^68^ (SCD) of the acyl chains of DPPC:DG-CDP(1:1) membrane (Figure 7a) and the distribution of the tilt angles between the lipid tails and the normal of the bilayer (Figure 7b) in the presence and absence of the DOX molecule have been computed from the molecular dynamics simulation. The presence of the DOX molecule slightly decreases the SCD and makes the tilt distribution a bit broader, meaning that the order of the lipid membrane slightly decreases. The tilt angle agrees well with the experimental value. The distribution of tilt angles presents two peaks because the angles for the lipids from the upper and lower layers are computed with respect to the same normal vector.

**Figure 7:**
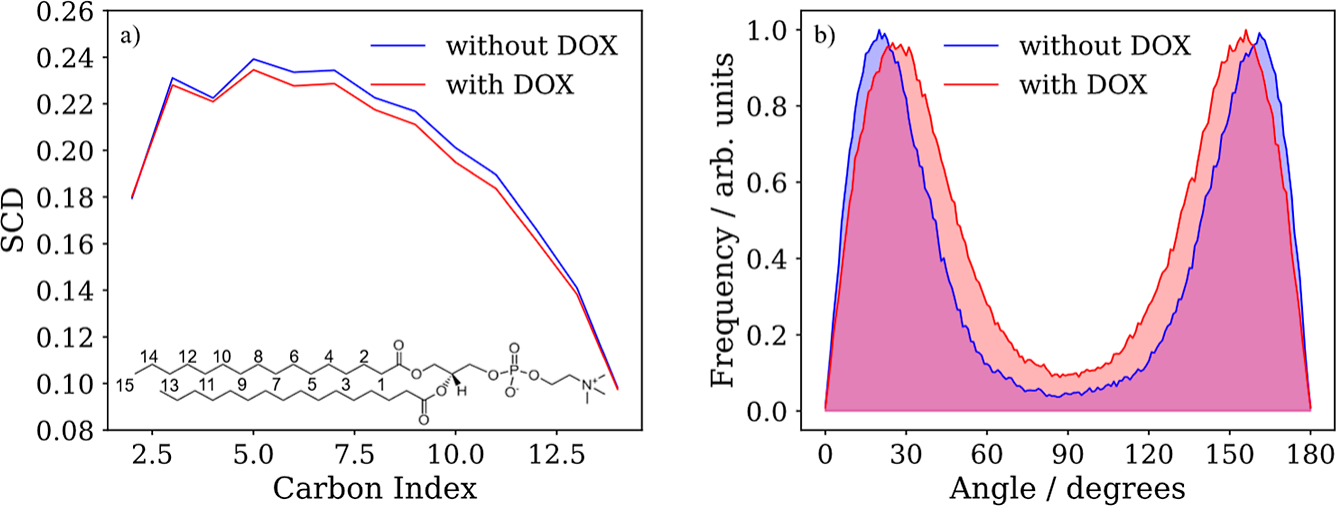
(a) Deuterium order parameter from molecular dynamics simulations of the acyl chains of the DPPC:DG-CDP(1:1) bilayer with (red) and without DOX (blue). (b) Distribution of the tilt angle of the DPPC:DG-CDP(1:1) membrane with (red) and without DOX (blue).

The computational results of the influence of DOX in the bilayer model, a small decrease in SCD suggesting a slight loss of order and a small increase of the tilt angle distribution broadness of acyl chains suggesting a slight decrease of the order, are the opposite of the experimental results. Results from both methods are within the precision of the respective techniques: ATR involves baseline corrections and deconvolutions, and the interactions in the molecular dynamics simulations are computed classically by a force field. Therefore, the combined experimental and computational tilt angles of the acyl chains indicate that the presence of DOX does not significantly affect the SCD of the lipid bilayer or the stability of the liposomes wall.

Figure 8c,d contains the polarized ATR spectra of Si-supported lipid bilayers in the region of ca. 1600 cm^–1^, at which C=O stretching and skeletal vibrations of the cytosine moiety and of DOX can be expected to present absorption bands. In order to analyze the different contributions, the transmission spectra of DOX and DPPC:DG-CDP(3:7) liposomes have been included. The assignments of the bands corresponding to the transmission spectrum of DOX in D_2_O solutions, in Figure 8a, that were made on the basis of DFT calculations, are given in Table S2. The spectrum shows 4 intense bands at 1725, 1616, 1589, and 1559 cm^–1^ corresponding to the aliphatic CO stretching (C_13_O), the two anthracene CO stretchings (C_5_O and C_12_O), and skeletal vibrations of the first ring. Table S2 also includes the assignment of absorption bands of the mixed-lipid liposomes, Figure 8b, made in a previous work.^17^ Briefly, C=O stretching of the acyl chains shows an absorption band at 1725 (solvated) and 1740 cm^–1^ (unsolvated). The rest of the bands in the spectral region in Figure 8b correspond to in-plane vibration of the cytosine moiety of DG-CDP.

**Figure 8:**
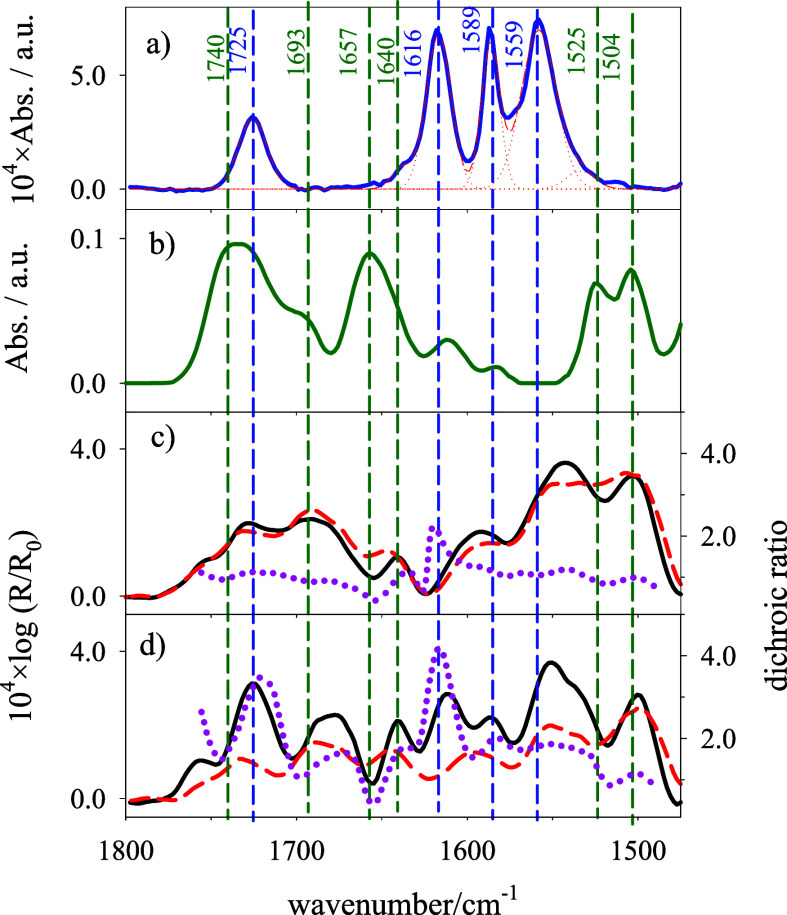
Transmission FT-IR spectra of (a) 0.1 mM DOX in D_2_O and (b) DPPC:DG-CDP(3:7) liposomes in D_2_O. (c,d) ATR spectra in the 1450–1800 cm^–1^ region of the DPPC:DG-CDP(3:7) bilayer supported on Si in contact with 0.1 M NaF/D_2_O in the absence (panel c) and presence (panel d) of DOX 100 μM, measured with light p (black solid line) and light s (dashed red line). Pink dotted lines represent the dichroic ratios. Blue and green vertical dashed lines represent the positions of absorption bands of DOX and nucleolipid, respectively.

The ATR spectrum of the lipid bilayer shows wider bands than the transmission spectra of the liposomes and a change in the relative intensities of the acyl C=O stretching at 1725 and 1740 cm^–1^. These phenomena suggest the presence of more intermolecular interactions of the polar heads, responsible for the absorption in this region, in the case of the supported bilayer than in the case of the liposomes.

In the presence of DOX in the supporting electrolyte, the ATR spectrum clearly shows the bands corresponding to DOX, although quite overlapped to the bands of the nucleolipid polar heads; therefore, it is difficult to separate different contributions. It can be observed that the position of IR absorption bands of DOX adsorbed on the bilayer is the same as in solution, indicating that the adsorption does not alter the acid–base and/tautomeric form of DOX. In the case of cytidine moiety, only the bands at 1505 and 1640 cm^–1^ are in “clean” parts of the spectrum in the presence of DOX. The band at 1505 cm^–1^ corresponds to the stretching vibrations of cytosine N3C4 and C4N7, with the transition dipole vector in the cytosine plane. As can be observed in Figure 8, the dichroic ratio, *R*, values of the band at 1505 cm^–1^ are close to 1, either in the presence or in the absence of DOX. These values of the dichroic ratio correspond to an angle between the transition dipole vector of the vibration and the normal direction of the bilayer of ca. 72°. This is in good agreement with the values obtained for the tilt angle of the same vibration in monolayers of the lipid mixture supported on Au(111) electrodes by PM-IRRAS (ca. 70°).^17^ Moreover, the fact that it does not change in the presence of DOX indicates that the orientation of the transition vector does not change much by the interaction with DOX. On the contrary, the dichroic ratio at 1640 and 1668 cm^–1^ clearly increases in the presence of DOX. Band corresponds to a coupled skeletal vibration of cytosine and ribose rings of cytidine moiety, with a high uncertainty in the direction of the transition dipole vector. The band at 1668 cm^–1^ has a principal contribution from the stretching vibration of cytosine CO group, and its transition dipole is oriented along the CO bond. According to the increase in the dichroic ratio, both transition dipoles become more perpendicular to the interphase in the presence of DOX. On the contrary, the presence of DOX does not affect significantly the position of the absorption IR bands corresponding to the cytosine moiety, on the contrary that expected for vibrations corresponding to molecular fragments involved in the interaction with DOX.

Regarding the DOX, absorption bands in Figure 8, all present high dichroic ratios, suggesting an orientation of the anthracene plane close to the normal direction of the bilayer. Moreover, the high dichroic ratio of the C_13_O stretching band at 1725 cm^–1^ suggests that the C_13_O bond is oriented close to the normal direction of the bilayer. On the other hand, the position of DOX bands in solution at 1616 cm^–1^ (C_5_O stretching), 1589 (C_12_O stretching), and 1559 cm^–1^ (skeletal ring 1) does not change when adsorbed on the bilayer. These are vibrations of the anthracene plane of DOX and the fact that their positions are not affected by the adsorption on the bilayer indicates that this DOX moiety does not participate in the interactions with the bilayer, in excellent agreement with the computational results, which concluded that the adsorption takes place by electrostatic interactions of the nonaromatic ring of DOX.

## Conclusions

Characterizing the nature of drug–lipid interactions is paramount to unveil the mechanism by which drugs can diffuse lipid membranes and, thus, to design improved drug delivery platforms. In this work, we investigate by means of experiments and theory the nature of the interactions of a model system: DOX is integrated into monolayers and bilayers formed by nucleolipids. Compression isotherms of Langmuir monolayers obtained in the absence and presence of DOX in the subphase indicate that DOX interacts only with the nucleolipid components of the lipid films containing DPPC and DG-CDP. Computer simulations confirm this conclusion, showing that adsorbed DOX is mainly located at the nucleoside fragments of the polar head of the nucleolipid. The adsorption of DOX is regulated by electrostatic interactions between the nonaromatic part of DOX and the polar heads and by stacking interactions between the aromatic moiety of DOX and the nucleobase.

Pseudocapacitance plots of Au(111) electrodes modified with a DPPC:DG-CDP(3:7) bilayer show lower values when DOX is present in the supporting electrolyte. This indicates that the lipid film becomes more isolating in the presence of DOX, diminishing the number and size of defects. The ATR spectra of supported DPPC:DG-CDP(3:7) planar bilayers on silicon, obtained with polarized radiation, provided the tilt angle of the acyl chains relative to the normal direction of the planar bilayer. The values obtained correspond to a liquid crystal phase and are in good agreement with the previous results obtained by PM-IRRAS for monolayers of the same composition. Molecular dynamics simulations provide close values for the tilt angle. The small changes observed for the average tilt angle in the presence of DOX, a small decrease from ATR or a small increase from computer simulations, suggest that the presence of DOX does not significantly affect the order of the lipid films.

The ATR spectral region at ca. 1600 cm^–1^ shows the bands of vibrations in the anthracene plane of adsorbed DOX at the same positions as in the spectrum in solution, suggesting that this fragment of DOX does not interact with nucleolipid polar heads, as the computer simulation results indicate. Moreover, the high dichroic ratios for these vibrations permit to conclude that the anthracene plane of DOX adopts a tilted orientation relative to the bilayer plane.

This work demonstrates the existence of electrostatic and stacking interactions between the anticancer drug DOX and the nucleoside fragments of the liposomes developed for its targeted delivery. These interactions do not alter the acid–base or tautomeric form of DOX. The combined methodology applied to the present study can serve as a reference for other studies of interactions between drug and carrier components.

## Supplementary Material



## Notes

RMSD of DPPC and DPPC:DG-CDP(1:1) bilayers with and without DOX; area per molecule calculated for DPPC and DPPC:DG-CDP(1:1) bilayers with and without DOX; elastic compression modulus obtained for monolayers of DPPC and DPPC:DG-CDP(3:7) at the air/water and at the air/0.1 M NaF interphases, in the presence and absence of 10 μM DOX in the subphase; insertion kinetics plots of DOX into DPPC:DG-CDP(3:7) monolayers; assignment of the IR absorption bands corresponding the CH stretching of the acyl chains; schematic representation of the phases involved in the ATR measurements; and assignment of the IR absorption bands corresponding to DOX and to the polar head region of DG-CDP (PDF)

## Notes

Dedicated to Prof. Jacek Lipkowski.

## Abbreviations

ATR: attenuated total reflection
DFT: density functional theory
DG-CDP: 1,2-dipalmitoyl-*sn*-glycero-3-cytidine diphosphate
DPPC: 1,2-dipalmitoyl-*sn*-glycero-3-phosphocholine
DPPS: 1,2-dipalmitoyl-*sn*-glycero-3-phosphoserine
DOX: doxorubicin
PM-IRRAS: photon-modulated infrared reflection–absorption spectroscopy
SCD: deuterium order parameter
