# Supplementary material for: Unveiling the Interactions of Doxorubicin with the Lipid Components of Liposomes for Its Delivery
Source: J Phys Chem B. 2025 May 6;129(19):4715–27. doi: 10.1021/acs.jpcb.5c00523 (PMC12135043; doi:10.1021/acs.jpcb.5c00523)
Supplement: Supplementary file 1 [file jp5c00523_si_001.pdf]

Unveiling the Interactions of Doxorubicin with Lipid Components of Liposomes for its Delivery.

Julia Alvarez-Malmagro[a‡], Lorena Ruano[b‡], María Cuartero-González[a], Juan J. Nogueira[b,c], Francisco Prieto-Dapena[a*]*

[a]Departamento de Química Física, Facultad de Química, Universidad de Sevilla, 41012, Sevilla, Spain;

[b]Departamento de Química, Facultad de Ciencias, Universidad Autónoma de Madrid, 28049, Madrid, Spain;

[c]Institute for Advanced Research in Chemistry (IAdChem), Universidad Autónoma de Madrid, 28049, Madrid, Spain;

Supporting Information

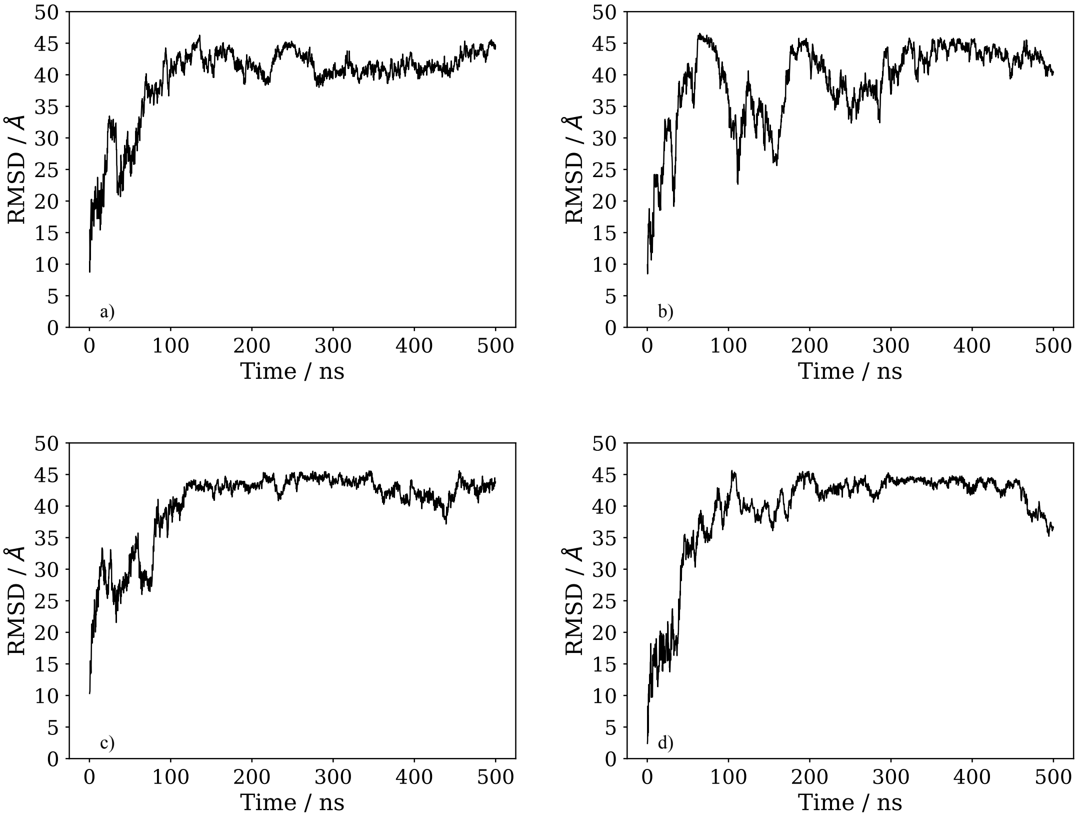

Figure S1. RMSD of DPPC membrane a) and DPPC:DG-CDP(1:1) bilayer b) without DOX and DPPC membrane c) and DPPC:DG-CDP(1:1) bilayer d) with DOX.

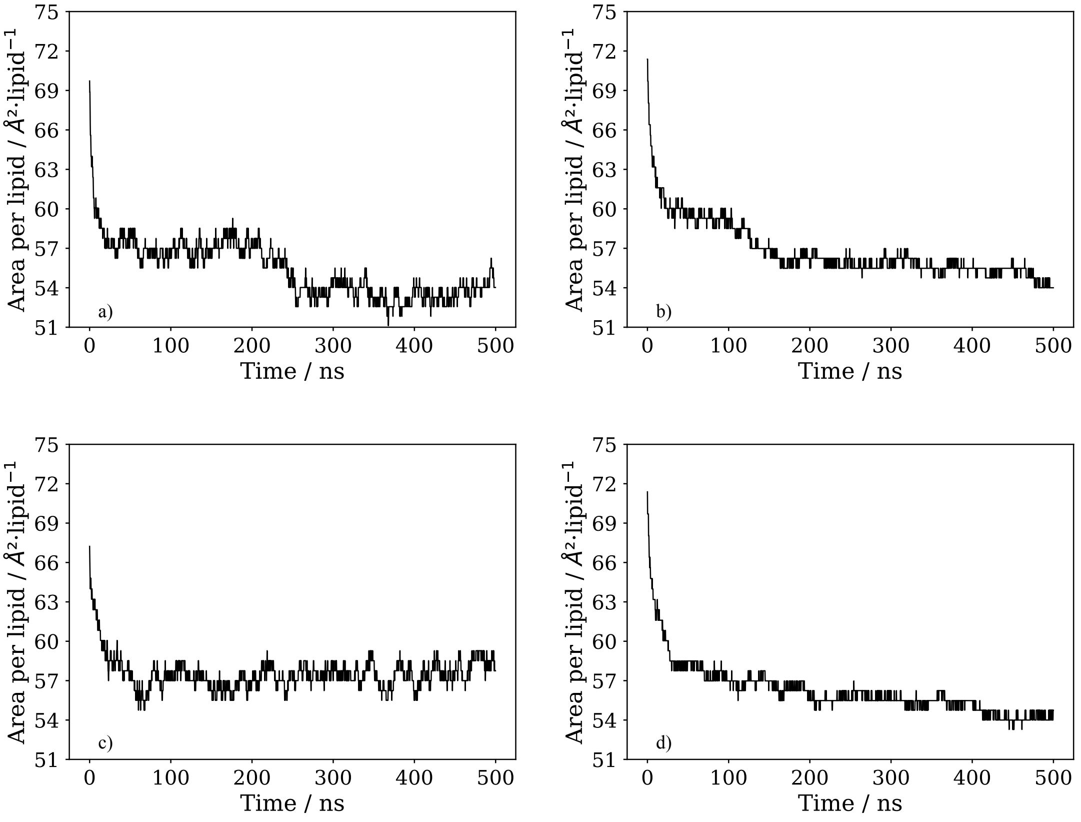

Figure S2. Area per lipid of DPPC membrane a) and DPPC:DG-CDP(1:1) bilayer b) without DOX and DPPC membrane c) and DPPC:DG-CDP(1:1) bilayer d) with DOX.

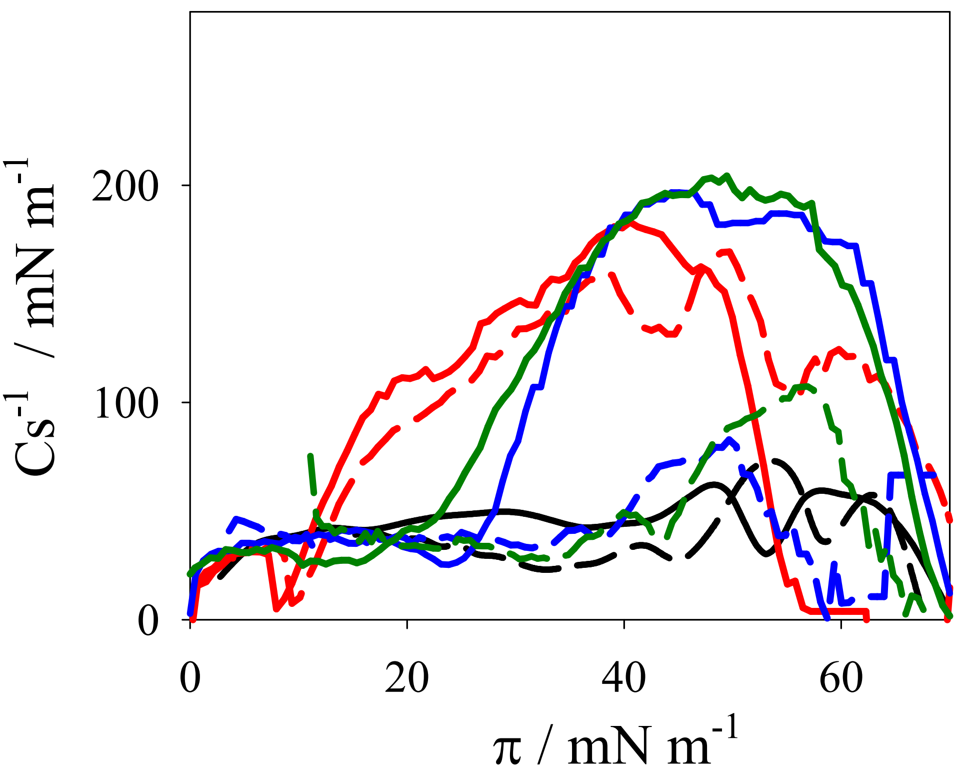

Figure S3.- Compression modulus (C_s^{-1}) calculated from the compression isotherms in Figure 1 with equation (1) :at the air/water interphase with monolayers of pure DPPC (red lines), pure DG-CDP (blue lines) and mixtures DPPC:DG-CDP (3:7, optimum mole composition) (green lines) and at the 0.1M NaF/air interphase (black lines) in the presence (dashed lines) and in the absence (solid lines) of DOX 10 μM in the subphase.

According to Davis and Rideal, [1] C_s^{-1} values below 70 mN m^{-1} correspond to a liquid expanded (LE) monolayer state, and values between 100-200 mN m^{-1} to liquid crystalline (LC) phase. The presence of DOX diminishes the maximum values of the compression modulus from c.a. 200 mN m^{-1} to 100 mN m^{-1} and 80 mN m^{-1} for the monolayers of pure DG-CDP and DPPC:DG-CDP(3:7), respectively.

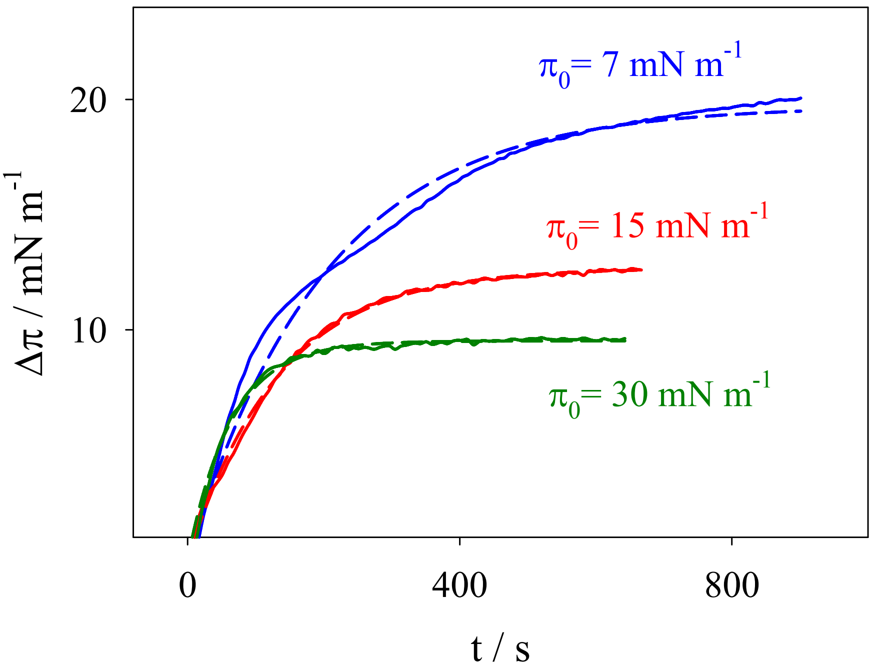

Figure S4.- Change in the surface pressure ($\Delta\pi=\pi-\pi_0$) at constant molecular area measured at the air/0.1 M NaF interphase at 24 °C with monolayers of mixtures DPPC:DG-CDP(3:7) as a function of the time after the injection of DOX 10 µM in the subphase. The initial surface pressure (π_0) values used are indicated in the plot. Dashed lines represent the theoretical plots obtained from the fitting to a pseudo first order kinetic model corresponding to equation (S1) [2]

$$\Delta\pi = \Delta\pi_{max}(1 - e^{-\beta t}) \qquad \text{(S1)}$$

The fitting of the plots in Figure S4 to equation (S1) provides, as adjustable parameters, the values of $\Delta\pi_{max}$, that represents the maximum surface pressure enhancement after the injection of DOX, and β, the pseudo-first order kinetic parameter that includes DOX concentration. [2] The representation of $\Delta\pi_{max}$ vs π_0, Figure S5, provide the *exclusion surface pressure*, from the abscissa intercept.

Table S1.- Assignment of the IR absorption bands of lipid acyl chains in the CH stretching region.

$\bar{\boldsymbol{\nu}}_{\boldsymbol{exp}}$ $(\boldsymbol{cm}^{-1})$	Assignment
2850	$\nu_{sim}(CH_2)$
2873	$\nu_{sim}(CH_3)$
2898	Fermi resonance
2921	$\nu_{asim}(CH_2)$

2936	Fermi resonance
2955	$\nu_{asim}(CH_3)$

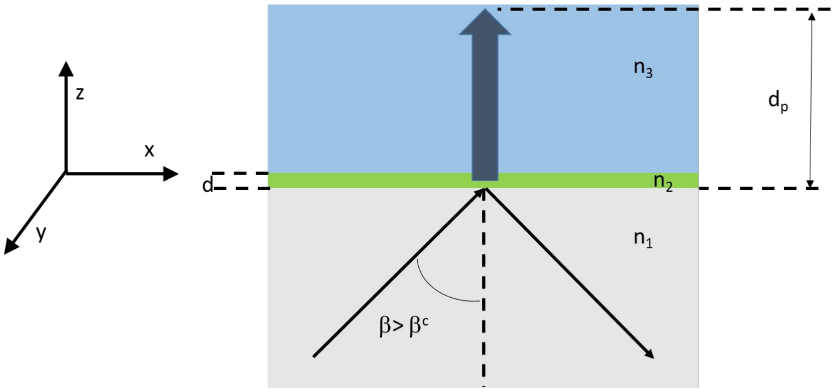

Figure S5. Schematic representation of the section of the interphase in the xz plane, including a thin layer of sample. n_1, n_2 and n_3 are the refraction indexes of the reflection media (Si), sample (lipid bilayer) and transmission media (electrolyte solution), respectively. β^c is the critical angle for total reflection. For the set up used in this work, n1:3.142, n2:1.44 and n3:1.33

Electric field intensities in x, y and z directions

$$E_x = \frac{2\cos(\beta)}{(1-n_{31}^2)^{0.5}[(1-n_{31}^2)sen^2(\beta)-n_{31}^2]^{0.5}} \quad \text{(S2)}$$

$$E_y = \frac{2\cos(\beta)}{(1-n_{31}^2)^{0.5}} \quad \text{(S3)}$$

$$E_z = \frac{2n_{32}^2\,\text{sen}(\beta)\cos(\beta)}{(1-n_{31}^2)^{0.5}[(1-n_{31}^2)sen^2(\beta)-n_{31}^2]^{0.5}} \quad \text{(S4)}$$

Where $n_{31} = {}^{n_3}/_{n_1}$ and $n_{32} = {}^{n_3}/_{n_2}$, with n_1, n_2 and $n_3$being the refraction indexes of the media 1 (Si), 2 (organic thin layer) and 3 (aqueous solution).

Table S2.- Assignment of the IR absorption bands of cytidine-5DP, 16:0 CDP:DG vesicles and DOX. Assignment of the PM-IRRAS absorption bands of 16:0 CDP:DG is also included

Cytidine-5DP[3–6]	16:0 CDP DG vesicles	16:0 CDP DG PM-IRRAS	DOX in D_2O solution
	1741 (νC=O)$_{acyl\ chain}$<br>1725 (νC=O)$_{acyl\ chain}$<br>1665 (νC=O)$_{cytosine}$	1668 (νC=O+ $\nu C_5=C_6$)	1725 (νC_{13}=O)
1654 (C+R)	1644 (C+R)	1643 (C+R)	
1613 ($\nu C_5=C_6$)	1606 ($\nu C_5=C_6$)	1614 ($\nu C_5=C_6$+ νC=O)	1616 (νC_5=O)
1581 (ν-ring)	1579 (ν-ring)		1589 (νC_{12}=O)<br>1559 Sq (ring1+δCH)
1524 (ν-ring)	1524 (ν-ring)	1527 (ν C4C5 + δCH)	
1503 (ν-ring)	1502 (ν-ring)	1501 (ν N3C4 + C4N7)	

References

(1) DAVIES, J. T.; RIDEAL, E. K. Chapter 5 - Properties of Monolayers. In *Interfacial Phenomena (Second Edition)*; DAVIES, J. T., RIDEAL, E. K., Eds.; Academic Press, 1961; pp 217–281. https://doi.org/https://doi.org/10.1016/B978-0-12-206056-4.50009-6.

(2) Marchenkova, M. A.; Dyakova, Y. A.; Tereschenko, E. Y.; Kovalchuk, M. V.; Vladimirov, Y. A. Cytochrome c Complexes with Cardiolipin Monolayer Formed under Different Surface Pressure. *Langmuir* **2015**, *31* (45), 12426–12436. https://doi.org/10.1021/acs.langmuir.5b03155.

(3) Starikov, E.B.; Semenov, M. A. IR-Spectroscopic and Quantum-Chemical Study of Hydration of Citidine-5'- Monophsphate Disodium Salts. *Zh.Fiz.Khim* **1988**, *62*, 2120–2126.

(4) Borah, B; Wood, J. L. The Cytidinium-Cytidine Complex: Infrared and Raman Spectroscopic Studies. *J Mol Struct* **1976**, *30*, 13–30.

(5) Mathlouthi, M; Seuvre, A. M. F.T.I.R and Laser-Raman Spectra of Cytosine and Cytidine. *Carbohydr Res* **1986**, *146*, 1–13.

(6) Kulihska, K.; Sarzyiiska, J.; Wiewiorowski, M. Differences in the Association Abilities in Aqueous Solutions of Cytidine , 2 '-Deoxycytidine and Their

Phosphate Salts Studied by Fourier Transform Infrared Spectroscopy. *Vib Spectrosc* **1991**, *1*, 277–286.
